# Efficient Day-and-Night NO_2_ Abatement by Polyaniline/TiO_2_ Nanocomposites

**DOI:** 10.3390/ma16031304

**Published:** 2023-02-03

**Authors:** Daniela Meroni, Melissa G. Galloni, Carolina Cionti, Giuseppina Cerrato, Ermelinda Falletta, Claudia L. Bianchi

**Affiliations:** 1 Dipartimento di Chimica, Università degli Studi di Milano, via Camillo Golgi 19, 20133 Milano, Italy; 2 Consorzio Interuniversitario Nazionale per la Scienza e Tecnologia dei Materiali (INSTM), via Giusti 9, 50121 Florence, Italy; 3 Dipartimento di Chimica, Università degli Studi di Torino, via Pietro Giuria 7, 10125 Torino, Italy

**Keywords:** polyaniline, titania, nanocomposites, NO_2_ abatement, air pollution, environmental remediation

## Abstract

Finding innovative and highly performing approaches for NOx degradation represents a key challenge to enhance the air quality of our environment. In this study, the high efficiency of PANI/TiO_2_ nanostructures in the NO_2_ abatement both in the dark and under light irradiation is demonstrated for the first time. Heterostructures were synthesized by a “green” method and their composition, structure, morphology and oxidation state were investigated by a combination of characterization techniques. The results show that the unique PANI structure promotes two mechanisms for the NO_2_ abatement in the dark (adsorption on the polymeric chains and chemical reduction to NO), whereas the photocatalytic behavior prevails under light irradiation, leading to the complete NOx degradation. The best-performing materials were subjected to recycling tests, thereby showing high stability without any significant activity loss. Overall, the presented material can represent an innovative and efficient night-and-day solution for NOx abatement.

## 1. Introduction

Air pollution today continues to pose a threat to health worldwide. Recently, the World Health Organization (WHO) has estimated that about 4.2 and 3.8 million deaths per year are due to the exposure to outdoor air pollution and to the household exposure to smoke from dirty cookstoves and fuels, respectively. In addition, about 91% of the world population lives in places where the air quality exceeds the WHO guideline limits [1]. All these updates suggest that a proper control of air pollutant emissions is required.

Among the main classes of air pollutants, nitrogen oxides (NOx) identify a family of binary compounds of nitrogen and oxygen, including nitric oxide (NO) and nitrogen dioxide (NO_2_) [2,3]. Both these species have been considered extremely harmful pollutants since 1952, when their role in the formation of photochemical smog was confirmed [4]. However, more in detail, NO, a colorless gas, is about four times less toxic than NO_2_, a reddish-brown gas [5,6].

In this drastic scenario, over the years numerous strategies have been developed and properly optimized with the final aim of decomposing NOx species. Among all the possibilities, their selective catalytic reduction by ammonia as a reducing agent (NH_3_-SCR) is one of the most studied and applied technologies [7,8,9], as well as absorption and adsorption routes [6,10]. However, from a practical viewpoint all these techniques suffer from several drawbacks, limiting their real application [3,8,11]. In this context, the ever more stringent legislative constraints related to NOx emissions are spurring the optimization of highly efficient techniques for NOx abatement [12]. Following these perspectives, innovative strategies based on the photocatalysis principles have emerged due to their efficiency, high sustainability and potential for integration in air purification systems [6,13,14,15,16]. 

For several years, titanium dioxide (TiO_2_) has been the most exploited photocatalytic system for a wide variety of applications, ranging from pollution abatement (in terms of water and air remediation) [17,18,19] to energy conversion [18,20]. TiO_2_ has gained research community attention because of some interesting features, such as wide availability, chemical stability, and low cost. It is characterized by a wide energy bandgap (3.2 eV for anatase, and 3.0 eV for rutile) [21,22,23] that makes it highly active in the ultraviolet region [24,25].

Limiting the attention on the NOx photodegradation, several works report the successful use of TiO_2_-based materials as photocatalysts [20,26,27,28,29,30,31]. While TiO_2_ displays good activity under UV-A, under LED light, pristine TiO_2_ is not able to reach the high value of NOx conversion. When promoted by metal species of photocatalytic interest (i.e., silver), it can produce enhanced activity under visible light, achieving up to 80% NOx conversion [32]. However, it has to be underlined that TiO_2_ remains inactive in the dark [33]. The performances of TiO_2_-based photocatalysts have been also largely investigated in outdoor experiments in streets, as summarized in different works [6,34,35]. In general, when TiO_2_-based photocatalysts are used as components of paints, asphalts and concretes, they are able to confer a unique activity in the NO photodegradation, thus demonstrating their successful application in several sectors [35]. In a more specific way, as examples, Qi et al. demonstrated that the addition of TiO_2_ to a polyacrylonitrile-styrene-acrylate resin results in a successful strategy to obtain an enhanced solar reflectance [36]. Moreover, Chen et al. realized a complete evaluation related to the NOx photodegradation on active concrete road surfaces in both laboratory and real scale application, demonstrating that TiO_2_-activated concrete road has good purifying effects toward NOx and the cost application was found to be very competitive [37]. However, if on the one hand the photodegradation tests carried out at laboratory scale are mostly promising, on the other hand the tests obtained for real applications are often conflicting and not always encouraging, making hard to draw general conclusions [38,39]. In fact, in real scenarios the different meteorological conditions (wind speed, humidity level, irradiation level, rainfall level, etc.), as well as the variable traffic intensity strongly affect the materials performances. Photocatalytic NOx abatement in real outdoor settings is known to be hampered by issues such as insufficient contact time and adsorption competition by air humidity. In this respect, a potential solution could be represented by a combination of an adsorbent and a photocatalyst, as here proposed.

Conducting organic polymers (COPs) have recently emerged as interesting materials in environmental remediation [40,41,42]. In general, they are employed as adsorbents for the removal of several types of pollutants (dyes [43,44], metals [45,46]) or as photosensitizers [47,48,49,50]. Among COPs, polyaniline (PANI) occupies a special place for its unique characteristics and, therefore, it can be considered a valid candidate to be used in the fields, where its interesting redox properties related to the presence of imino-quinoid/amino-benzenoid groups can make the difference [42,51,52,53,54,55,56,57,58]. So far, concerning NOx, COPs have been largely studied to fabricate gas sensors [59,60,61]. Indeed, they can be coupled with other materials (e.g., carbon nanotubes, CNTs, TiO_2_, SnO_2_, ZnO), obtaining interesting systems composed of three elements. This strategy has been adopted to overcome the limitations related to binary composites (e.g., weak and slow interaction between the sensing material and the target gas and low response) [62,63,64,65,66,67]. By way of example, Yun et al. prepared a PANI/MWCNT/TiO_2_ composite sensor described by modest selectivity to NO, but with excellent reproducibility [68]. Then, Xu et al. fabricated a SnO_2_/ZnO/PANI composite thick film able to give high sensor response at 180 °C to NO_2_ (35 ppm) in only 30 s [69]. However, a common limitation of these systems is their poor stability and sensitivity [70].

Following these premises, in this work, for the first time, we present the results obtained in the NO_2_ abatement under both dark and light irradiation by PANI/TiO_2_ nanocomposites properly projected. PANI/TiO_2_ heterostructures were fabricated by an innovative green UV-assisted procedure and characterized by a combination of physicochemical techniques (PXRD, UV-vis, FTIR, SEM-EDS, BET, TG) [56]. The amount of PANI in the final composite was accurately dosed to the final enhancement of the night-time activity without affecting photocatalytic properties. The role of PANI in the NO_2_ abatement was also investigated based on its physicochemical characteristics. The best materials were subjected to recycling tests to demonstrate their stability in the working conditions.

## 2. Materials and Methods

### 2.1. Sample Preparation

PANI-TiO_2_ heterostructures were synthesized according to a previously reported procedure [56,71].

In brief, Evonik P25 TiO_2_ (anatase: rutile 80: 20, 50 m^2^/g) was added under vigorous stirring to 290 mL of an aqueous solution prepared by dissolving 1 g of *N*-(4-aminophenyl)aniline in HCl 0.1 M. The use of HCl is crucial not only to solubilize the reagent (*N*-(4-aminophenyl)aniline), but also for the progress of the reaction that requires acidic conditions and to obtain the final PANI in its conducting form (half-oxidized and half-protonated). Then, the suspension was irradiated with a UV halogen lamp (500 W, 30 mW/cm^2^) for 2 h and subsequently stirred in the dark for further 4 h at room temperature. TiO_2_ particles guarantee the formation of PANI oligomers and act as catalysts during the subsequent step that consists in the addition of a suitable amount of 30% H_2_O_2_ aqueous solution to carry out oxidative polymerization reaction. The reaction mixture was then stirred in the dark and at room temperature for 1 day. Finally, the precipitate was collected by filtration, washed with water and acetone and dried at room temperature. Samples with different TiO_2_ contents were synthesized by varying the (*N*-(4-aminophenyl)aniline: TiO_2_: H_2_O_2_ molar ratios, namely 1.0:0.4:3.0, 1.0:0.4:1.0 and 1.0:0.8:1.0, which will be named PANI-TiO_2__15%, PANI-TiO_2__31% and PANI-TiO_2__67%, respectively, where the percentage is related to the amount of the polymer in the final composites.

For the sake of comparison, pure PANI samples, prepared according to the literature syntheses, were also investigated as the references. The first, labeled as PANI_ref1, was prepared according to a conventional synthetic approach [51,72] starting from 0.04 M aniline aqueous solution acidified by HCl (aniline/HCl molar ratio = 0.1) and using K_2_S_2_O_8_ as oxidant. The reaction was carried out at 0 °C, adopting an aniline/K_2_S_2_O_8_ molar ratio of 0.67. The precipitate was collected by filtration after 8 h, then washed with water and acetone and dried at room temperature.

A second reference (named PANI_ref2) sample was synthesized from *N*-(4-aminophenyl)aniline using H_2_O_2_ as the sole oxidant in the presence of FeCl_3_ (*N*-(4-aminophenyl)aniline/Fe^3+^ = 4000 wt/wt), able to catalyze the oxidative polymerization reaction [73,74]. In brief, 2.35 mL of 30% H_2_O_2_ aqueous solution was added to 250 mL of an aqueous solution prepared by dissolving 1 g of *N*-(4-aminophenyl aniline) in HCl 0.1 M. Then, 0.15 mL of a 5 g/L FeCl_3_ aqueous solution was added. The precipitate was collected by filtration after 24 h, washed with water and acetone and dried at room temperature.

### 2.2. Sample Characterization

Powder X-ray diffraction (PXRP) was performed on a Philips PW 3710 Bragg–Brentano goniometer equipped with a graphite-monochromatic Cu Kα radiation source, scintillation counter, 1° divergence slit, 0.2 mm receiving slit and a 0.04° Soller slit system. Diffractograms were collected at a nominal X-ray power of 40 kV and 40 mA, in a 2θ range between 10° and 80°.

Thermogravimetric analyses (TGA) were collected on a TGA/DSC 3+ Mettler Toledo instrument equipped with a 70 μL alumina crucible. Measurements were carried out between 30 and 900 °C with a heating rate of 5 °C/min and in air.

Specific surface area values were determined from adsorption isotherms of N_2_ in subcritical conditions, measured using a Coulter SA3100 instrument (Beckman Life Sciences, Los Angeles, CA, USA) by the Brunauer–Emmett–Teller (BET) model.

UV-vis absorption spectra of the samples, dissolved in DMF with and without the HCl addition, were recorded in 200–1000 nm range on a Shimadzu UV-2600 UV-vis spectrophotometer (Kyoto, Japan).

Fourier Transform infrared (FTIR) spectra were measured on a PerkinElmer Spectrum 100 spectrometer (Waltham, MA, USA), working in attenuated total reflectance mode in 400–4000 cm^−1^ range.

The morphologic characterization was carried out without any sample pretreatment using a scanning electron microscope, operating with a Field Emission source (model TESCAN S9000G, (Overcoached, Germany); Source: Schottky type FEG; Resolution: 0.7 nm at 15 keV (in In-Beam SE mode) and equipped with EDS Oxford Ultim Max (operated with Aztec software 6.0).

### 2.3. NO_2_ Abatement

A total of 50 mg of each material was suspended in 5 mL isopropanol and sonicated by an ultrasonic bath to obtain a uniform dispersion. After 10 min, the mixture was deposited on a glass plate (3.3 cm × 11.5 cm) and dried in air overnight to obtain a thin film. Then, the glass plate was placed inside a 20 L Pyrex cylindrical batch reactor and NO_2_ gas abatement tests were performed. They were carried out at room temperature for 3 h in the dark and under both LED (350 mA, 9–48 V, 16.8 W, 2900 lx) and UVA (λ_max_ = 365 nm, 100 W/m^2^) irradiation. The initial concentration of NO_2_ was 500 ± 50 ppb. An ENVEA AC32e directly connected to the reactor measured the NOx (NO and NO_2_) concentration at 30, 60 and 180 min by chemiluminescence principle. Photolysis tests indicate 10% pollutant degradation after 3 h of UVA light irradiation.

### 2.4. Reusability Tests

PANI_ref1, bare TiO_2_ and PANI-TiO_2__31% were selected to be tested for four consecutive tests to evaluate their stability. The samples were prepared as described in Section 2.1. The tests were carried out at room temperature, exposing the material to 500 ppb of NO_2_. PANI_ref1 was maintained in the dark for 3 h, whereas bare TiO_2_ and PANI-TiO_2__31% were maintained in the dark for 1 h and then irradiated by UVA light for other 2 h. The chemiluminescence detector monitored NO, NO_2_ and NOx concentration at 30, 60 and 180 min. After each test, the material was removed from the reactor, left in air for 12 h and then used for the subsequent cycle.

## 3. Results and Discussion

All the synthesized materials were characterized by several techniques (TG, BET, PXRD, SEM-EDS, FTIR and UV-vis) and used for the NO_2_ abatement, as described below.

### 3.1. Materials Characterization

PANI-TiO_2_ composites with different TiO_2_ contents (from 15 to *ca.* 67%) were prepared varying the reactant molar ratios. 

**Figure 1:**
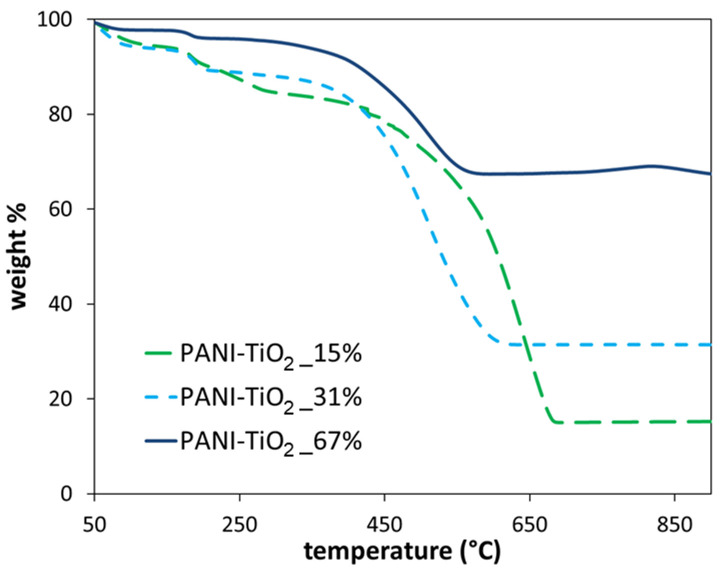
TGA curves of the PANI-TiO_2_ composites.

The actual composition, determined by TG analyses in air and expressed as weight percentage, is reported in Table 1, whereas the relative TG curves of the pristine PANIs and PANI-TiO_2_ composites are reported in Figure S1 and Figure 1.

Physisorbed water content was determined from the weight loss at around 100 °C, whereas the HCl amount was related to the weight loss at 150–200 °C and the polymer content was estimated from the weight loss starting around 350 °C, assigned to the polymer backbone degradation. The remaining fraction at 700 °C was attributed to TiO_2_. Interestingly, the thermal stability of the polymer backbone is affected by the TiO_2_ content, as higher oxide amounts weaken the polyaniline inter-chain interactions [75].

**Figure 2:**
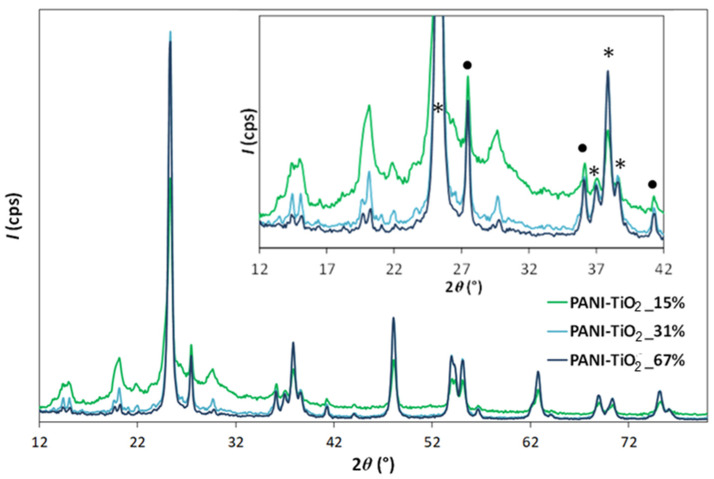
Diffractograms of the PANI-TiO_2_ composites. Inset: zoomed in 12–42° region (peaks attributed to anatase and rutile TiO_2_ are marked with a star and a circle, respectively).

The different PANI content is also appreciable in the PXRD patterns (Figure 2) from the relative intensity of the peaks attributed to the TiO_2_ polymorphs (anatase and rutile) and to the ES-I phase of the polymer [76] (Figure 2, inset). Moreover, decreasing the PANI content leads to a more ordered arrangement of the polymer chains, i.e., a higher crystallinity of the PANI phases, as appreciable from the better resolved peaks.

**Figure 3:**
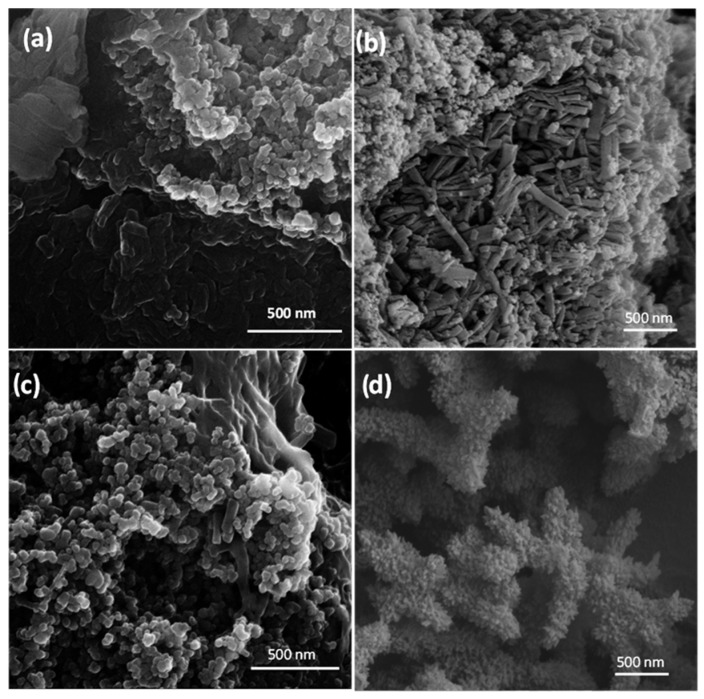
SEM images of (**a**) PANI-TiO_2__15%, (**b**) PANI-TiO_2__31%, (**c**) PANI-TiO_2__67% and (**d**) PANI_ref1.

The enhanced crystallinity of the PANI-TiO_2_ heterostructures with higher oxide content pairs with the larger specific surface area values that are greatly promoted compared to the PANI reference materials (Table 1 and Figures S2 and S3). These differences between the nanocomposites and pristine PANIs can be traced back to the morphologies observed by SEM analyses. While the PANI_ref2 displays a compact morphology (Figure S4), PANI-TiO_2_ composites are composed of a matrix of PANI nanorods and aggregates of rounded oxide nanoparticles (Figure 3). Among them, the PANI-TiO_2__15% sample presents a less porous structure (Figure 3a) with PANI rods that look fused, explaining the lower surface area. PANI_ref1 displays an intermediate surface area, which could be related to its coral-like structure, displaying micrometric rods covered by nanometric spikes (Figure 3d).

The different structural features of the synthesized materials can be associated with the diverse synthetic mechanisms involved. In fact, the traditional synthesis of PANI_ref1 consists in a two-steps reaction, involving the production of active radical species (aniline and its dimers) during the step 1, followed by a chain elongation process (step 2) [55]. The possibility to control the first step permits obtaining well organized structures characterized by high surface areas and porous morphologies. Similarly, the synthesis of PANI/TiO_2_ composites follows the same approach. However, as shown in Table 1, in this case the control of the radical species’ production is correlated to the TiO_2_ quantity. In fact, for low amount of oxide particles, materials with poor characteristics are obtained (low value of surface area and more compact morphology). On the contrary, when the first step of the reaction occurs in the presence of larger amount of TiO_2_, the properties of the final composites improve. It is supposed that the first step of the reaction consists in the UV-mediated PANI oligomer formation on the TiO_2_ surface and that, during the second step, the oligomers’ polymerization occurs [71].

On the contrary, the reaction that leads to the production of PANI_ref2 does not permit a fine control of the radicals formation, because the oxidative polymerization of *N*-(4-aminophenyl)aniline is too fast to be controlled, leading to a decrease in the structural characteristics of the synthesized product [55].

**Figure 4:**
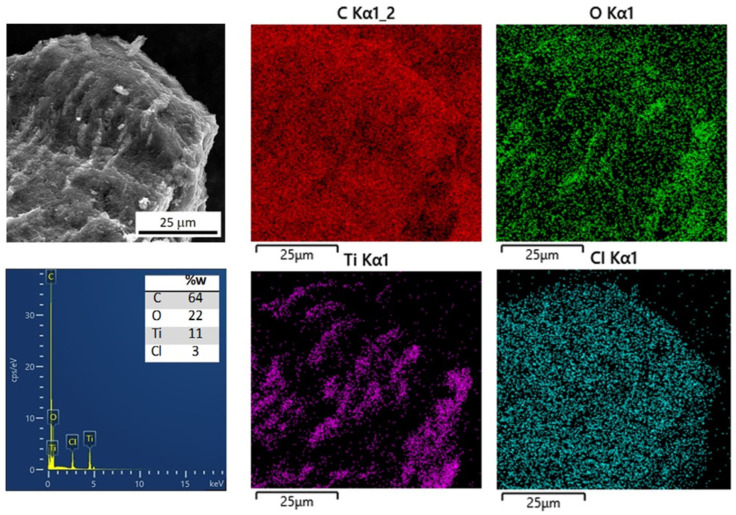
SEM image (top left-hand section) and EDX mapping of PANI-TiO_2__31% relative to the four elements revealed by EDX analysis (bottom left-hand section; inset: elemental composition from EDX analysis reported as weight percentage).

EDX mapping of the PANI-TiO_2_ composites confirms the non-uniform distribution of the oxide nanoparticles in the polymer matrix. In particular, the TiO_2_ particles seem to be localized along the polymer fibers (Figure 4).

**Figure 5:**
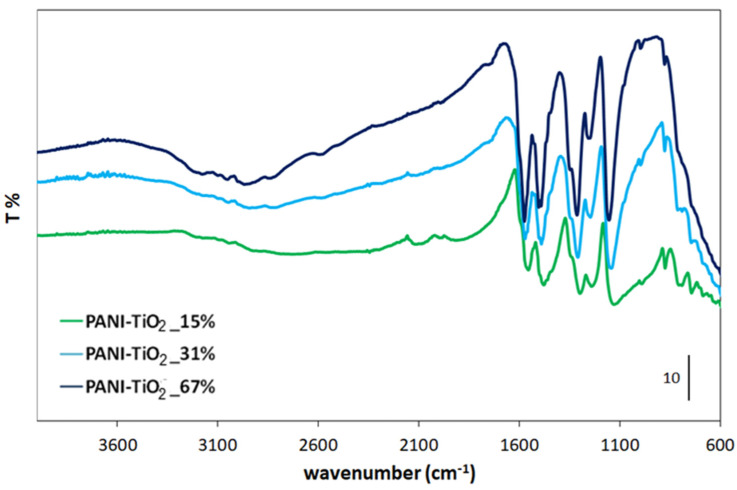
FTIR spectra of the PANI-TiO_2_ composites.

FTIR spectra (Figure 5) confirmed the formation of PANI in its emeraldine form, as appreciable from the similar relative intensity of the band of the quinoid ring C=C stretching (1570 cm^−1^) and of the stretching mode of the benzenoid ring (1490 cm^−1^) [51]. The band at about 1300 cm^−1^ is attributed to the C-N bending vibration in aromatic amines, whereas those at 1070 cm^−1^ and 870 cm^−1^ are related to the in-plane and out-of-plane bending modes of C-N. The stretching mode of N=Q=N (Q = quinoid ring) is responsible for the band at 1000 cm^−1^. Similar results were obtained for the two pristine PANI samples (Figure S5). The absence of signals in the 3600–3700 cm^−1^ region, characteristic of the free hydroxyl groups of TiO_2_, suggests a complete coating of TiO_2_ particles with the PANI matrix.

**Figure 6:**
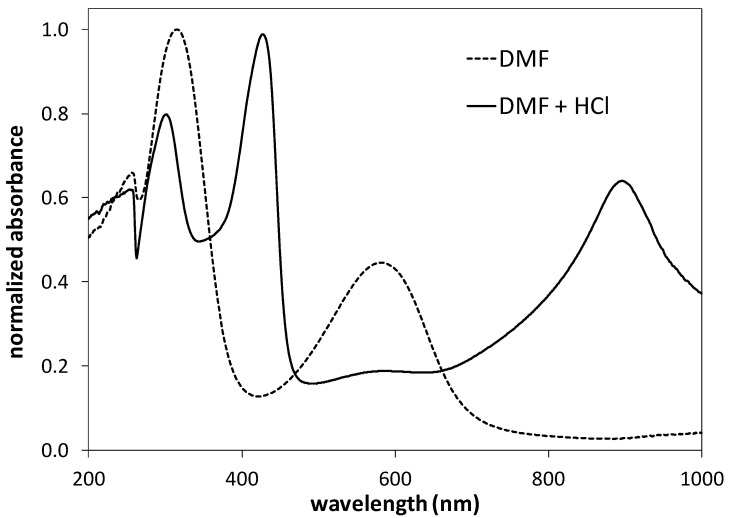
UV-vis spectra of PANI-TiO_2__31% acquired in DMF and in DMF acidified with HCl.

This observation is also supported by the UV-vis spectra of the samples dissolved in DMF (Figure 6), where the signals of both the oxidized and reduced part of the polymer are appreciable. As an example, the UV-vis spectrum of PANI-TiO_2__31% is shown in Figure 6. In particular, the π − π* transition of benzene is observed at ca. 310 nm, whereas the band at 580 nm can be attributed to the azaquinoid moieties π − π* transition band. Upon the HCl addition, the characteristic bands of emeraldine salt are observed, testifying the acid-base properties of the polymer: the signal around 310 nm is due to benzene rings, whereas the band around 430 nm is attributed to π − polaron and polaron − π* transitions, and the band at 900 nm has been related to polymer conformation changes, conjugation extension or to the de-aggregation of polymer chains in solution [77].

In contrast, the two UV-vis spectra of the two pristine PANIs show an important difference (Figure S6), strongly affecting the activity of the materials toward the NO_2_ abatement. In fact, comparing the intensity of the two main peaks, at ca. 310 nm and 600 nm, the second one, associated to quinone rings, is more intense for PANI_ref1, if compared to PANI_ref2, suggesting a greater number of imino-quinone rings in the chains on the polymer.

### 3.2. NO_2_ Abatement

**Figure 7:**
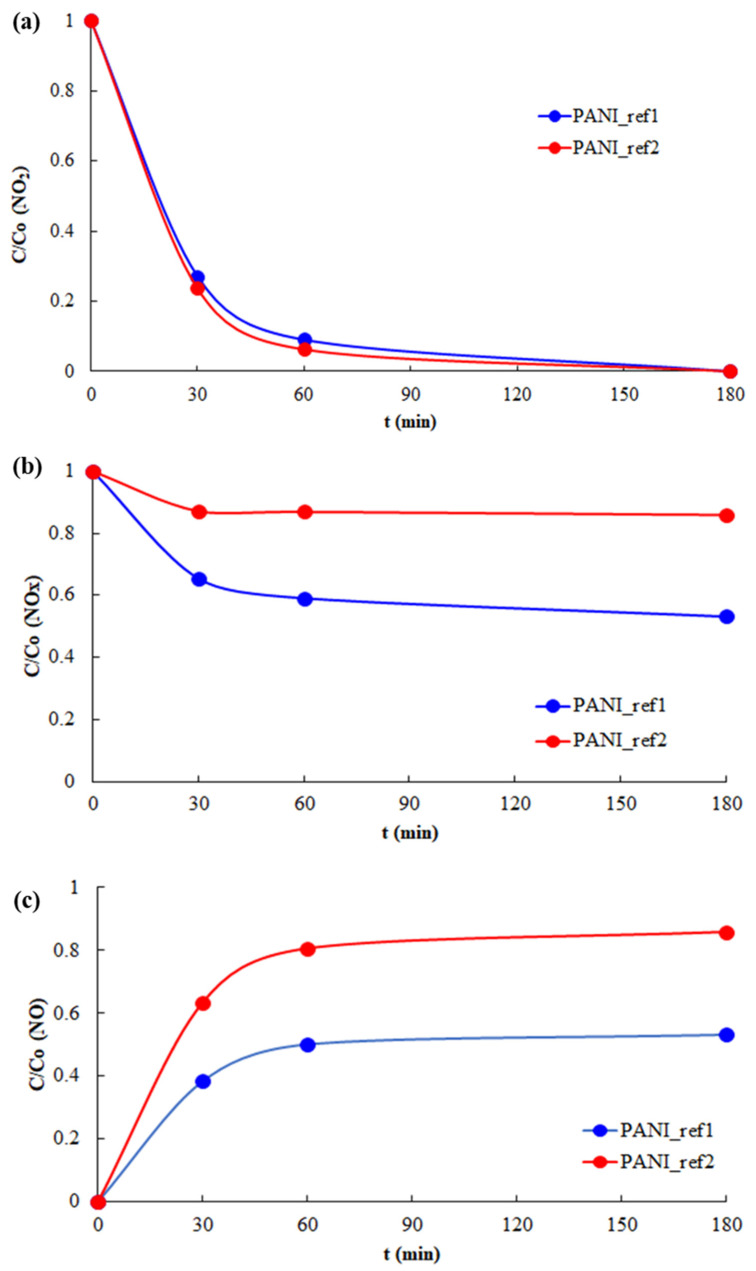
(**a**) NO_2_ and (**b**) NOx removal efficiency and (**c**) NO production as a function of time for PANI_ref1 and PANI_ref2 in dark.

All the synthesized materials were tested for the NOx abatement, exploiting the different characteristics of the components: redox properties and adsorption features of the PANI material and photocatalytic activity of TiO_2_. Figure 7 shows the results obtained when pristine PANI samples are used as active materials in dark conditions. In conditions, it was also evaluated that pristine TiO_2_ leads to a 5% of NOx adsorption.

**Scheme 1:**
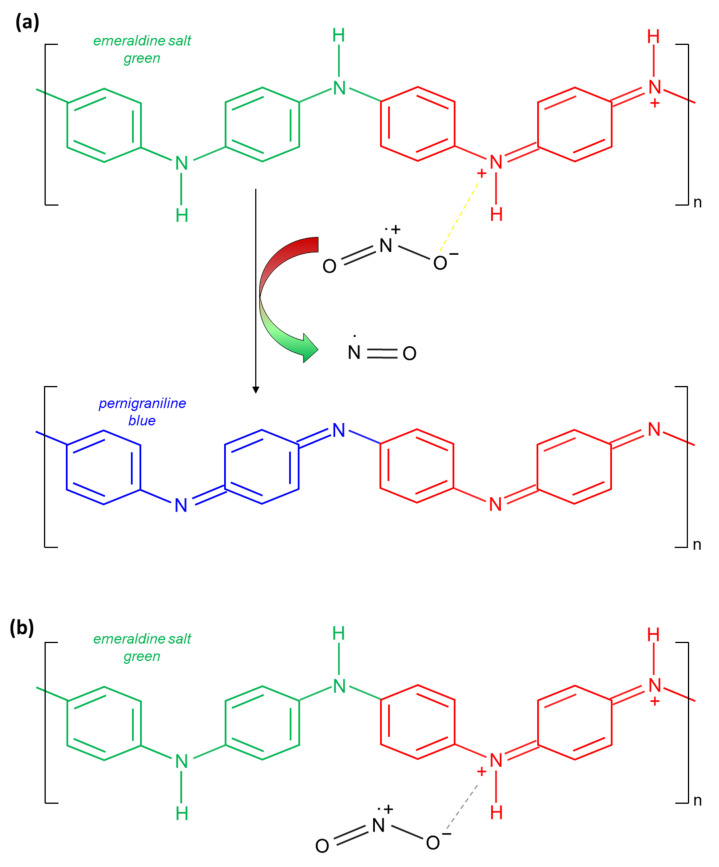
(**a**) NO_2_ chemical reduction and (**b**) NO_2_ adsorption by PANI.

Both PANI_ref1 and PANI_ref2 exhibit comparable activity in the NO_2_ abatement, reaching about 100% removal in 3 h (Figure 7a). If, at first, this extraordinary result could be attributed to the adsorption capability of the materials, already demonstrated for the VOC reduction in air matrix [55], the results of NOx abatement (Figure 7b) suggest otherwise. In fact, PANI_ref1 leads to the removal of 47% of the initial NOx, whereas PANI_ref2 reaches only 14% abatement. On the other hand, the NO analyses (Figure 7c) display a gradual increase in the NO amount during the tests. Based on these outcomes, it is possible to affirm that for the NOx removal, PANI acts by two simultaneous different paths: NO_2_ adsorption and chemical reduction in NO_2_ to NO, as described in Scheme 1.

The chemical reduction in NO_2_ to NO can be reasonably associated to the redox properties of the PANI chains. In fact, as described in Scheme 1, the amino-benzenic units are able to reduce NO_2_ to NO-producing imino-quinones units. In air, these latter are quickly regenerated maintaining the materials’ activity. As reported in the literature [78], NO_2_ is a strong electron acceptor gas able to carry out oxidation reactions. So far, the adsorption ability of PANI has been only highlighted, affirming that, at room temperature, NO_2_ is strongly adsorbed on the polymer, needing a proper thermal treatment for the material regeneration. Here, for the first time, we demonstrate that PANI acts through both NO_2_ adsorption and chemical reduction to NO that simultaneously permit the NO_2_ abatement.

It is worth noting that, although both the materials exhibit very similar performances in the NO_2_ removal, they differ in the preferential mechanism employed.

In fact, if, on the one hand, PANI_ref1 acts through both approaches, converting 54% NO_2_ to NO and adsorbing 46% of the initial NO_2_, on the other hand, PANI_ref2 proceeds almost exclusively by NO_2_ reduction, reducing 86% NO_2_ into NO and adsorbing only 14% of the initial gas. It is important to highlight that, unlike NO_2_, NO is not an irritant gas and much less toxic than NO_2_ [79]. No major role is attributed to an oxidation of inhaled NO into NO_2_ in the lungs since, after inhalation, NO is eliminated faster than it is oxidized NO [80].

The different behavior of the two polymers can be attributed to their difference in specific surface area values and oxidation level of the polymeric chains, as described above in Table 1 and Figure S6.

The attempts carried out to characterize the used materials by UV-vis spectroscopy did not show any evident differences compared to the fresh ones. In fact, the low NO_2_ initial concentration (50 ppb) and the fast internal redox chemical rearrangement between imino-quinone and amino-benzenic units do not permit to evidence a change in the polymer structure.

The test carried out under light irradiation (both UVA and LED) led to similar results (Figures S7 and S8), confirming that, in photocatalytic processes, pristine PANI does not act as a photocatalyst, but as a photosensitizer [81].

PANI/TiO_2_ nanocomposites showed a behavior intermediate between that of PANI and that of bare TiO_2_. In fact, when exposed to NO_2_ gas in the dark, they maintained the same activity of pristine PANI_ref2, removing almost completely the gaseous pollutant (Figure S9a). In contrast, concerning the NOx abatement, the performance of the materials is strictly dependent on the polymer content, increasing with the amount of PANI in the composites. Passing from PANI-TiO_2__15% to PANI-TiO_2__67%, the NOx abatement ranges from 35% to 50% (Figure S9b). On the contrary, the percentage of NO_2_ converted into NO follows the opposite trend: PANI-TiO_2__15% > PANI-TiO_2__31% > PANI-TiO_2__67% (Figure S9c), attributing to the increase in the polymer content this property of the heterostructures. As expected, in the dark, TiO_2_ does not show any activity in the NO_2_ abatement.

**Figure 8:**
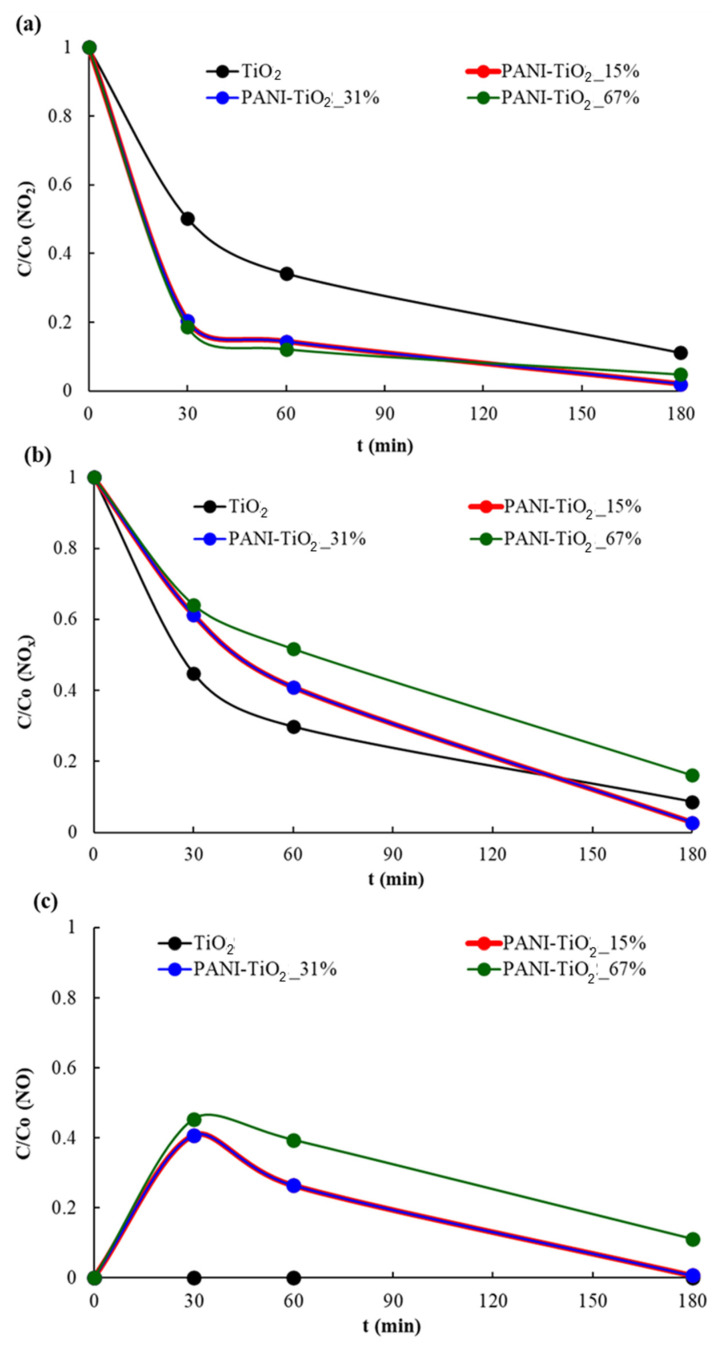
(**a**) NO_2_ and (**b**) NOx removal efficiency and (**c**) NO production as a function of time under UVA light.

When exposed to UVA light irradiation, PANI/TiO_2_ nanocomposites exhibit superior activity compared to the two bare components (PANI and TiO_2_), as reported in Figure 8.

PANI/TiO_2_ nanocomposites maintain a high capability toward the NO_2_ abatement that was similar to PANIs and greater than the performance of bare TiO_2_. In fact, all the heterostructures can remove more than 90% NO_2_ in 1 h, whereas the result with pristine TiO_2_ settles at 70% in the same conditions (Figure 8a). Moreover, concerning the NOx abatement, the heterostructures characterized by higher TiO_2_ content (PANI-TiO_2__15% and PANI-TiO_2__31%) lead to full NOx removal in 3 h, whereas when PANI content exceeds a certain amount (PANI-TiO_2__67%), the NOx degradation drops to 85% (Figure 8b). Finally, all nanocomposites in the first 30 min of light irradiation, acting by the chemical reduction path typical of PANI, convert 55–60% of initial NO_2_ to NO (Figure 8c). However, continuing with the irradiation, all the heterostructures are also able to degrade NO. The degradation percentage depends on the materials’ composition, leading to full NO removal in 3 h for the composites with higher TiO_2_ content (PANI-TiO_2__15% and PANI-TiO_2__31%) and 90% NO abatement for the photocatalyst more reachable in the polymer (PANI-TiO_2__67%).

As reported in the literature [82], the photocatalytic NOx degradation consists in a gradual oxidation of both NO and NO_2_ to HNO_3_ or NO_3_^−^ depending on the reaction conditions. However, in the presence of a certain amount of water droplets in the air (e.g., rain or artificial sprays of water), NO_2_ can undergo disproportionation, leading to HNO_3_ and NO that in air is quickly re-oxidized to NO_2_.

Although PANI is known to be a very promising photosensitizer able to extend the photoactivity of TiO_2_ in the visible part of the electromagnetic spectrum, the photocatalytic tests carried out under visible light irradiation showed that, under these conditions, all the nanocomposites maintain the activity of the polymeric component without any improvement in the photocatalytic properties (Figure S10). This is associated with the low TiO_2_ content of the heterostructures, if compared with other similar PANI/TiO_2_ materials employed in the photodegradation of pollutants [83].

### 3.3. Reusability Tests

PANI_ref1 and PANI-TiO_2__31% were selected to test their stability on the basis of the reusability tests. Each material was tested for four consecutive cycles, as reported above.

The reusability results obtained in the NO_2_ and NOx abatement and NO formation in the dark using PANI_ref1 as active material are reported in Figure S11, confirming the high stability of the polymer that exhibits the same behavior for all the performed tests.

As expected, bare TiO_2_ particles are completely inactive in the first 60 min, when the UVA light is off, whereas they quickly lead to NOx and NO_2_ photodegradation under light irradiation (Figure S12). The NO degradation is not reported, but the concentration was zero after light irradiation.

**Figure 9:**
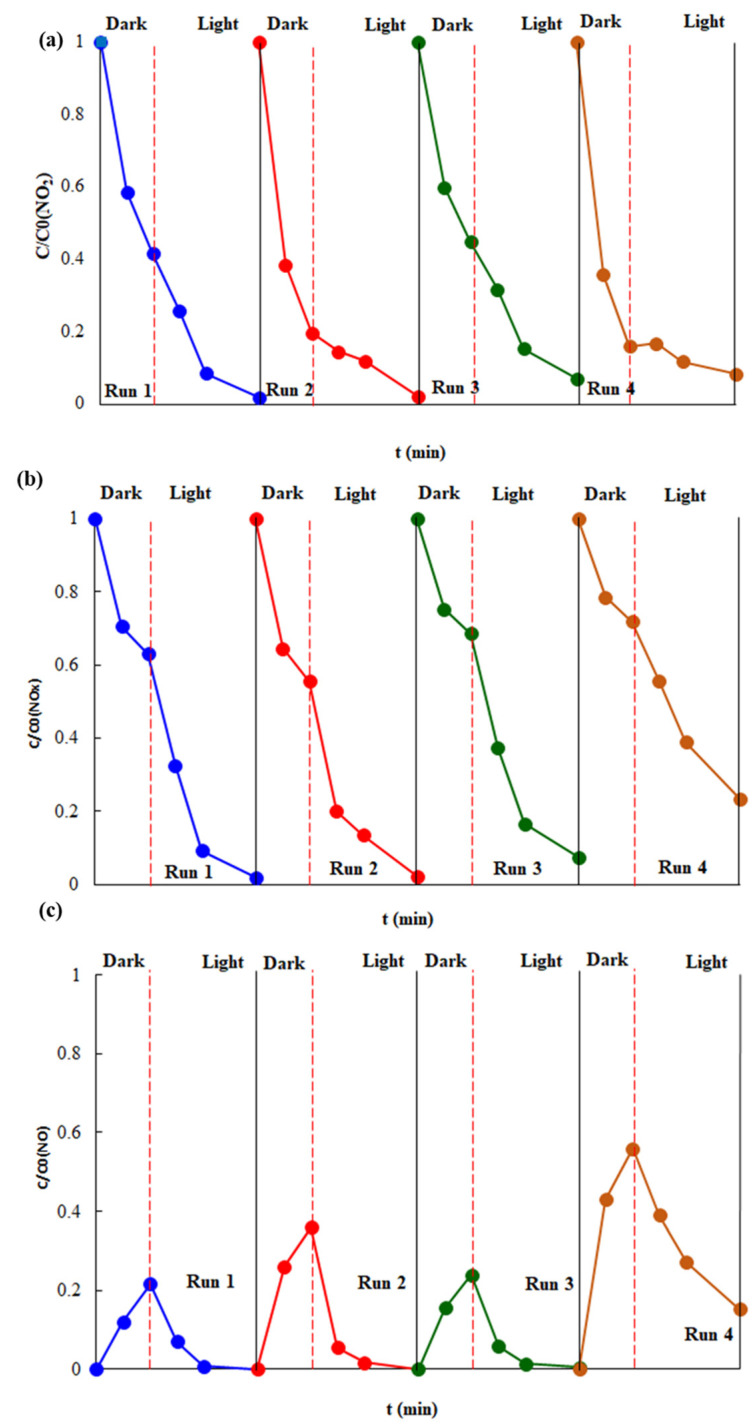
Recycle tests carried out with PANI-TiO_2__31% under dark and UVA irradiation: (**a**) NO_2_ and (**b**) NOx removal efficiency and (**c**) NO production.

Figure 9 shows the results of the reusability tests performed by PANI-TiO_2__31%.

PANI-TiO_2__31% exhibits very high stability for all the recycle tests. It is able to degrade about 60% NO_2_ in the dark, converting only 20% of the initial gas to NO (Figure 9a). Under light irradiation, its photocatalytic properties prevail, leading to quite complete NOx degradation (Figure 9b,c).

## 4. Conclusions

In this work, PANI/TiO_2_ nanocomposites were properly synthesized by a green UV light-driven oxidative polymerization and used for NO_2_ abatement. For the first time, the double role of the PANI matrix in the NO_2_ removal by both adsorption and chemical reduction to NO was demonstrated. The first mechanism (adsorption) can be correlated to the van der Walls interaction as well as to the formation of hydrogen bonds between the polluting gas and the polymer’s protonated amino and imino groups. Moreover, these organic groups (in particular -NH_2_) are able to strongly interact with polar species such as NO_2_ thanks to electrostatic dipole–dipole forces resulting in enhanced adsorption. In contrast, the second one (chemical reduction) affects the amino-benzenic units of PANI that are easily oxidized to the corresponding imino-quinone groups. The double mechanism ensures the total removal of NO_2_ in dark conditions but at the same time the NO accumulation. However, exploiting the photocatalytic properties of the heterostructures, when UVA light is turned on, all the nanocomposites are able to complete the NOx photodegradation in 3 h.

The high stability of the materials, tested through four consecutive cycles, makes them attractive for further investigation for the realization of innovative and efficient night-and-day surfaces to be applied for indoor and outdoor environments.

## Figures and Tables

**Table 1:** Weight percentage of PANI, water, HCl and TiO_2_ in the synthesized samples determined from TG analyses, and specific surface area from BET model of each PANI-based material.

Sample	Sample Composition (TGA)				S_BET_ (m^2^/g)
	% PANI	% TiO_2_	% H_2_O	% HCl	
PANI-TiO_2__15%	70	15	5	10	6
PANI-TiO_2__31%	58	31	6	5	48
PANI-TiO_2__67%	29	67	3	1	47
PANI_ref1	82	-	8	10	20
PANI_ref2	85	-	5	10	3

## Data Availability

The data that support the plots within this paper are available from the corresponding author on reasonable request.

## Supplementary Materials

The following supporting information can be downloaded at: https://www.mdpi.com/article/10.3390/ma16031304/s1, Figure S1: TGA curves of the pristine PANI_ref1 and the PANI_ref2; Figure S2: N_2_ adsorption isotherms in 0 < *p*/*p*^0^ < 0.2 for the samples; Figure S3: PXRD of PANI¬_ref1 and PANI_ref2; Figure S4: SEM of PANI_ref2; Figure S5: FTIR spectra of PANI_ref1 and PANI_ref2; Figure S6: UV-vis spectra of PANI_ref1 and PANI_ref2; Figure S7: (a) NO_2_ and (b) NOx removal efficiency and (c) NO production as a function of time for PANI1 and PANI2 under UVA light; Figure S8: (a) NO_2_ and (b) NOx removal efficiency and (c) NO production as a function of time for PANI1 and PANI2 under LED light; Figure S9: (a) NO_2_ and (b) NOx removal efficiency and (c) NO production as a function of time for TiO_2_ and PANI/TiO_2_ nanocomposites in the dark; Figure S10: (a) NO_2_ and (b) NOx removal efficiency and (c) NO production as a function of time for TiO_2_ and PANI/TiO_2_ nanocomposites under LED light; Figure S11: Recycle tests carried out with PANI_ref1 under dark: (a) NO_2_ and (b) NOx removal efficiency and (c) NO production; Figure S12: Recycle tests carried out with TiO_2_ under UVA irradiation: (a) NO_2_ and (b) NOx removal efficiency.
