# Supplementary material for: Efficient Day-and-Night NO2 Abatement by Polyaniline/TiO2 Nanocomposites
Source: Materials (Basel). 2023 Feb 3;16(3):1304. doi: 10.3390/ma16031304 (PMC9920043; doi:10.3390/ma16031304)
Supplement: Supplementary file 1 [file materials-16-01304-s001.zip › materials-2142633-supplementary.pdf]

Efficient Day-and-Night NO_2 Abatement by Polyaniline/TiO_2 Nanocomposites

Daniela Meroni [1,2], Melissa G. Galloni [1,2], Carolina Cionti [1], Giuseppina Cerrato [3], Ermelinda Falletta [1,2,*] and Claudia L. Bianchi [1,2]

[1] Dipartimento di Chimica, Università degli Studi di Milano, via Camillo Golgi 19, 20133 Milano, Italy
[2] Consorzio Interuniversitario Nazionale per la Scienza e Tecnologia dei Materiali (INSTM), via Giusti 9, 50121 Florence, Italy
[3] Dipartimento di Chimica, Università degli Studi di Torino, via Pietro Giuria 7, 10125 Torino, Italy
* Correspondence: ermelinda.falletta@unimi.it; Tel.: +39-02503114410

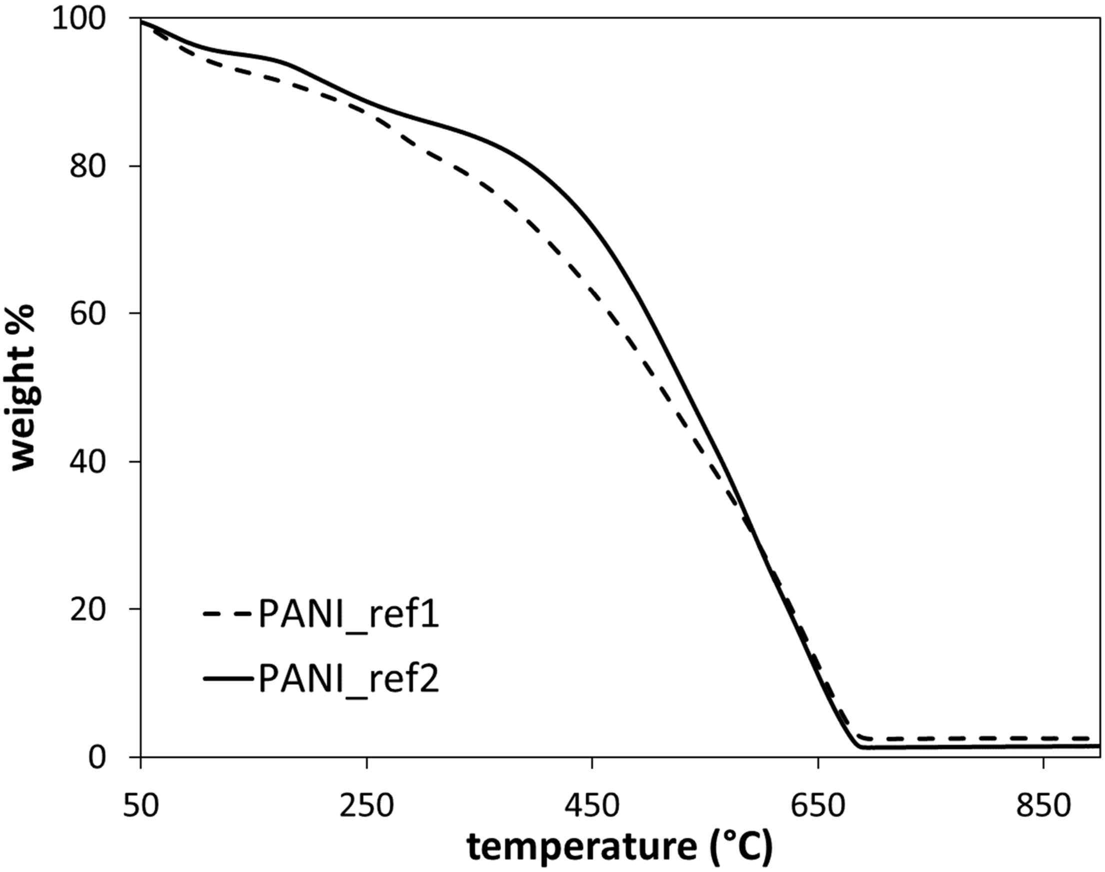

Figure S1: TGA curves of the pristine PANI_ref1 and the PANI_ref2.

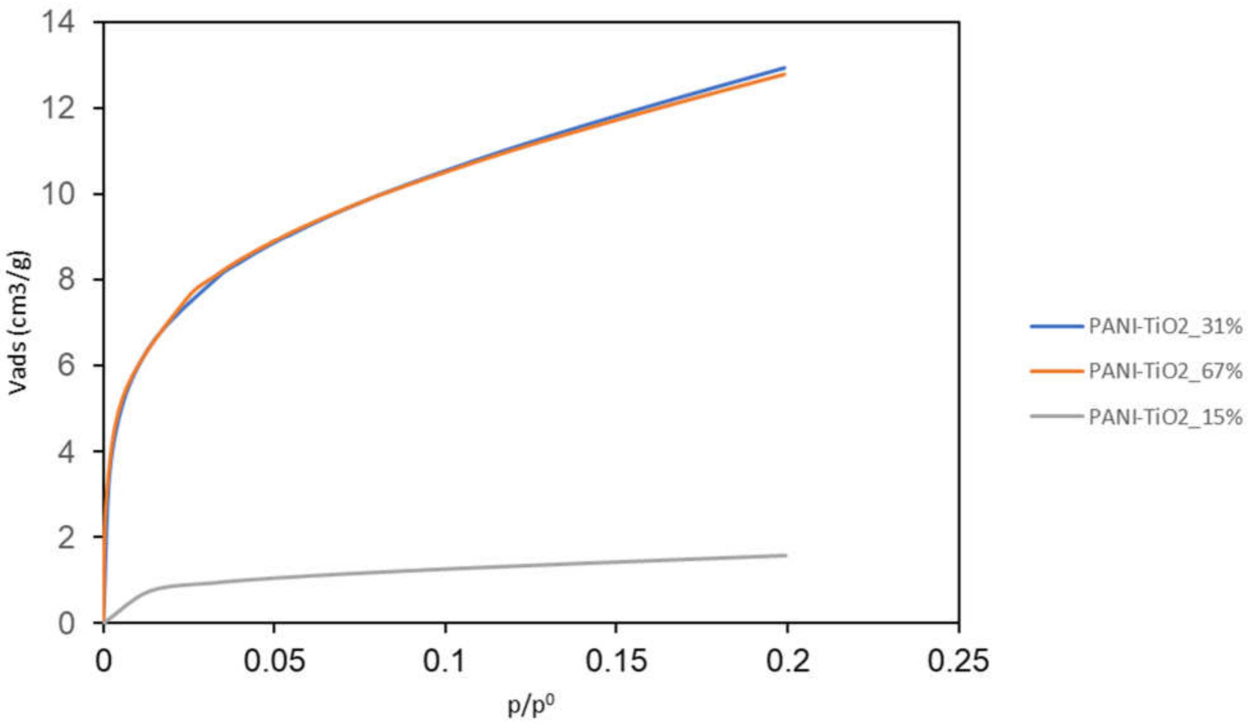

Figure S2: N_2 adsorption isotherms in $0 < p/p^0 < 0.2$ for the samples.

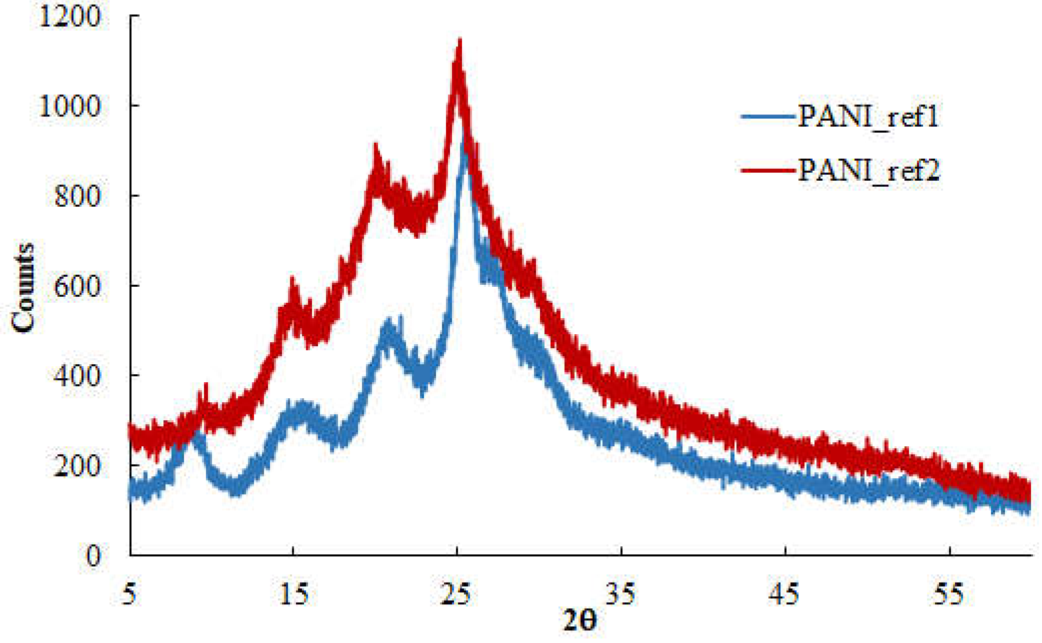

Figure S3: PXRD of PANI_ref1 and PANI_ref2.

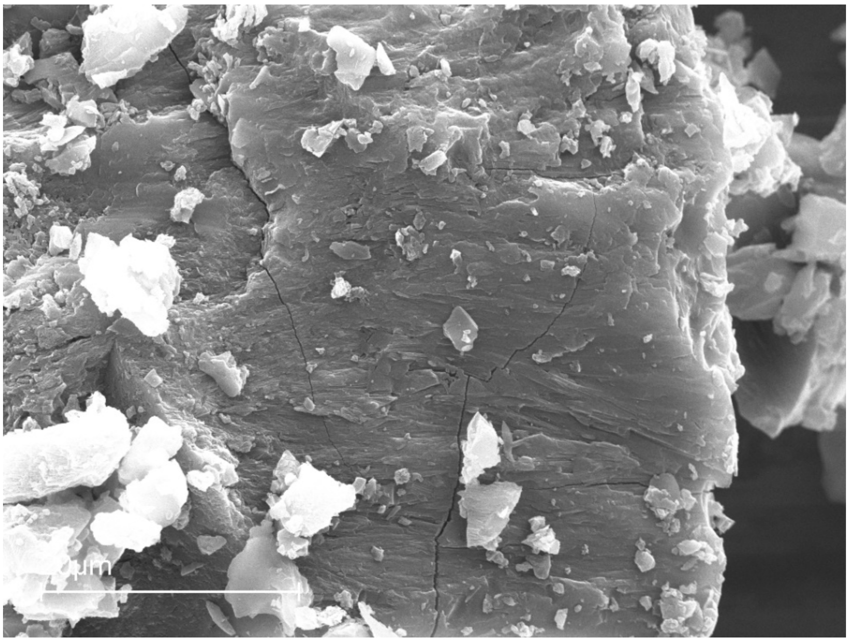

Figure S4: SEM of PANI_ref2.

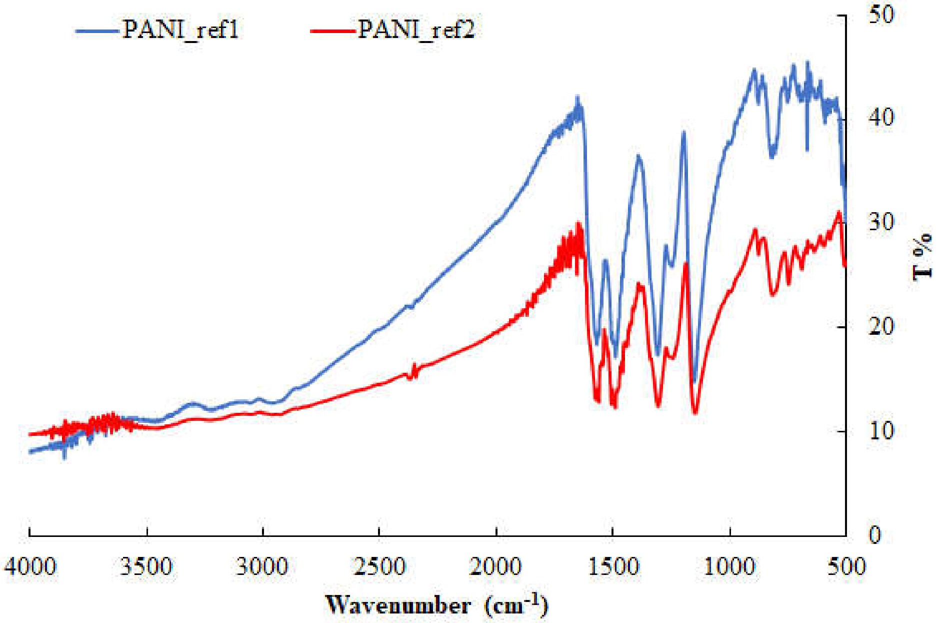

Figure S5: FTIR spectra of PANI_ref1 and PANI_ref2.

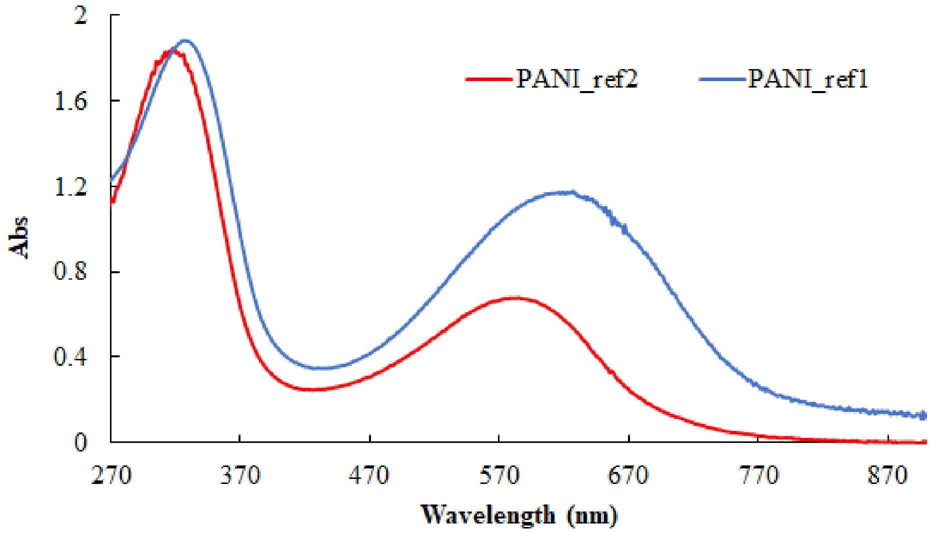

Figure S6: UV-vis spectra of PANI_ref1 and PANI_ref2.

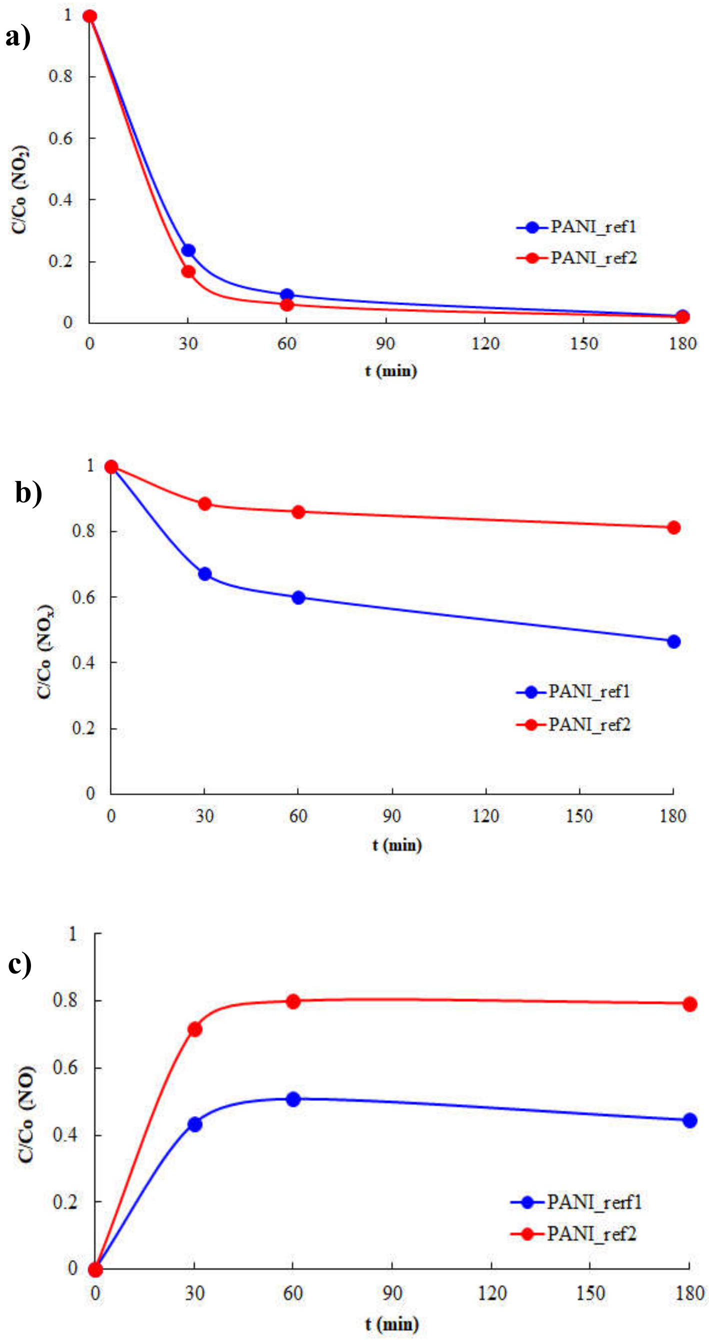

Figure S7: (**a**) NO_2 and (**b**) NOx removal efficiency and (**c**) NO production as a function of time for PANI1 and PANI2 under UVA light.

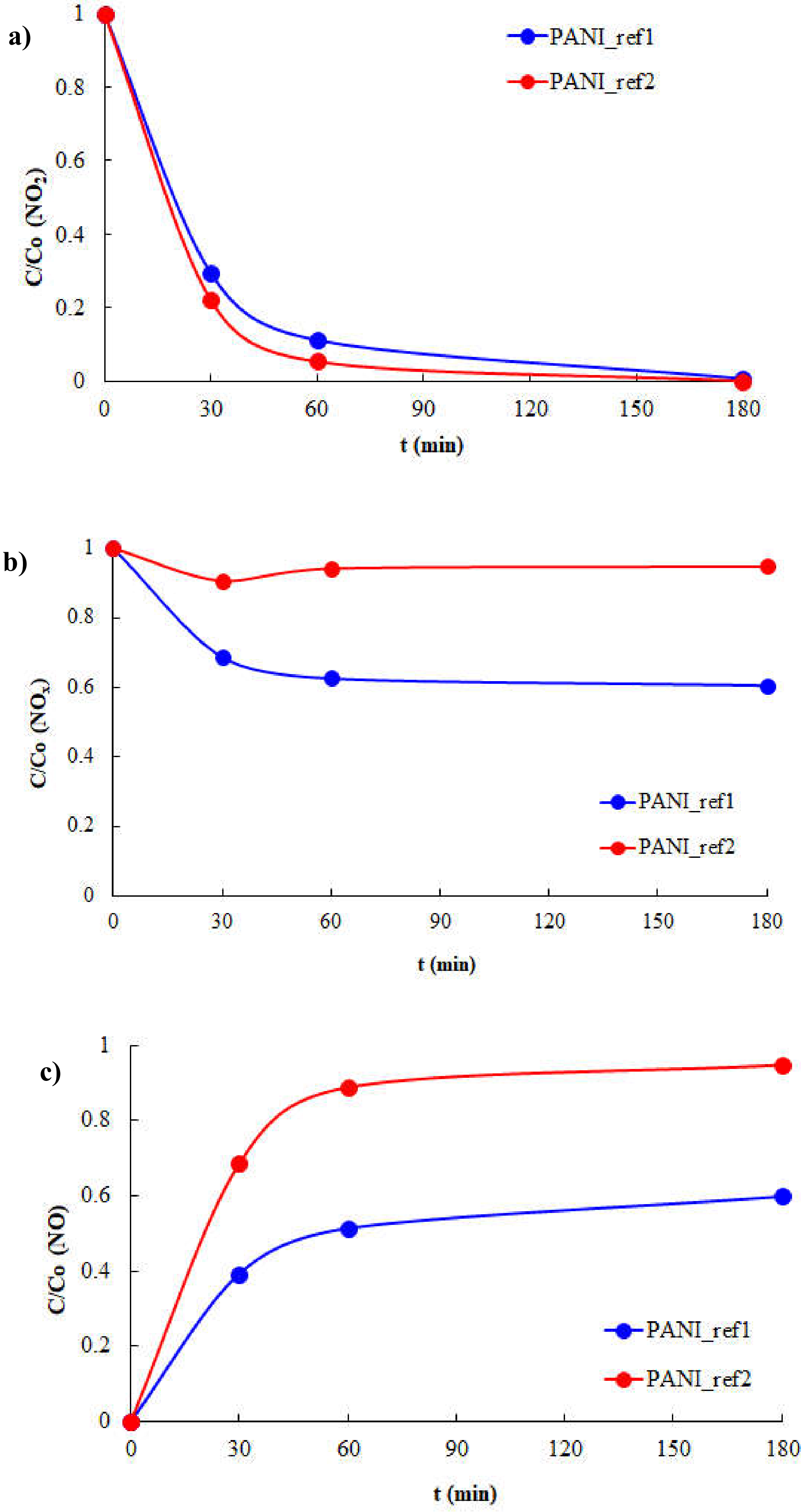
a)
PANI_ref1
PANI_ref2
C/Co (NO_2)
t (min)
b)
C/Co (NO_x)
t (min)
PANI_ref1
PANI_ref2
c)
C/Co (NO)
t (min)
PANI_ref1
PANI_ref2

Figure S8: (**a**) NO_2 and (**b**) NOx removal efficiency and (**c**) NO production as a function of time for PANI1 and PANI2 under LED light.

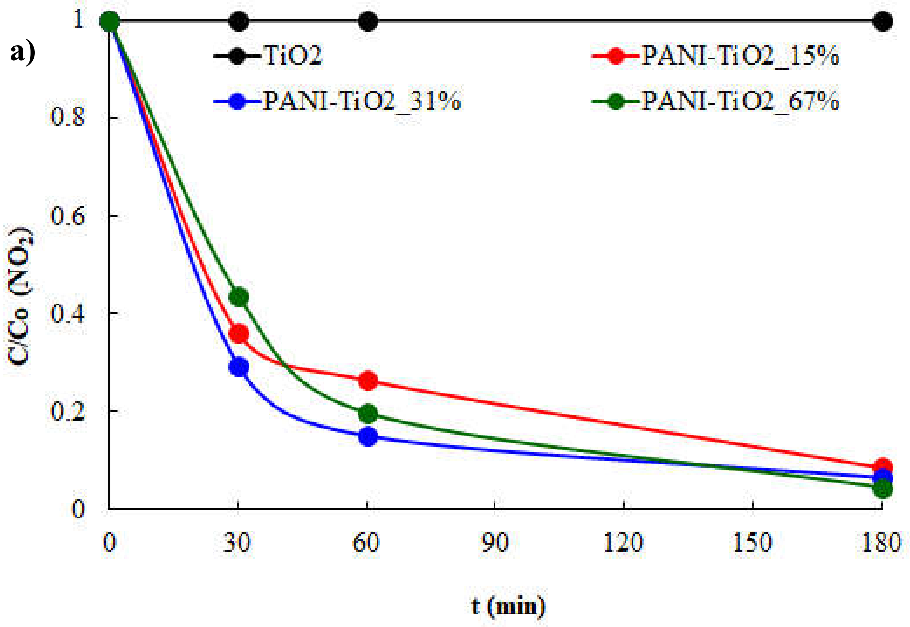

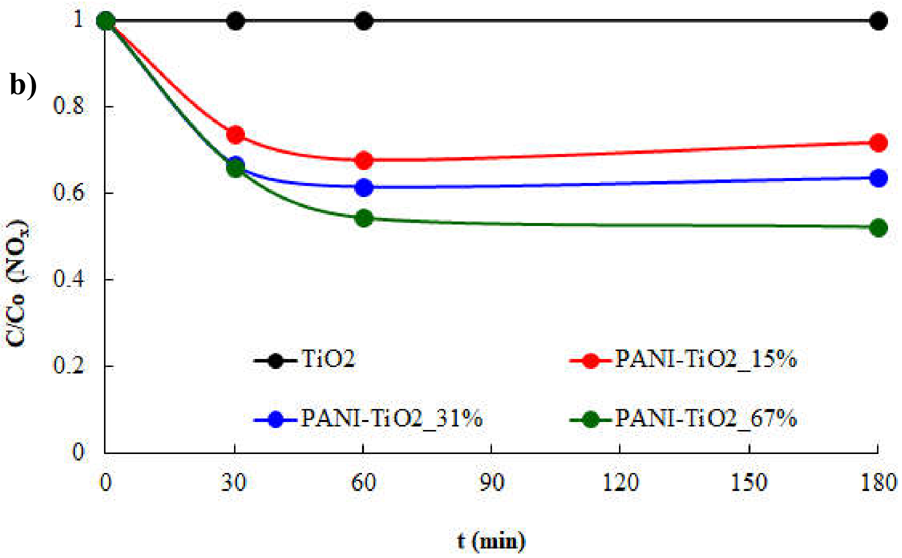

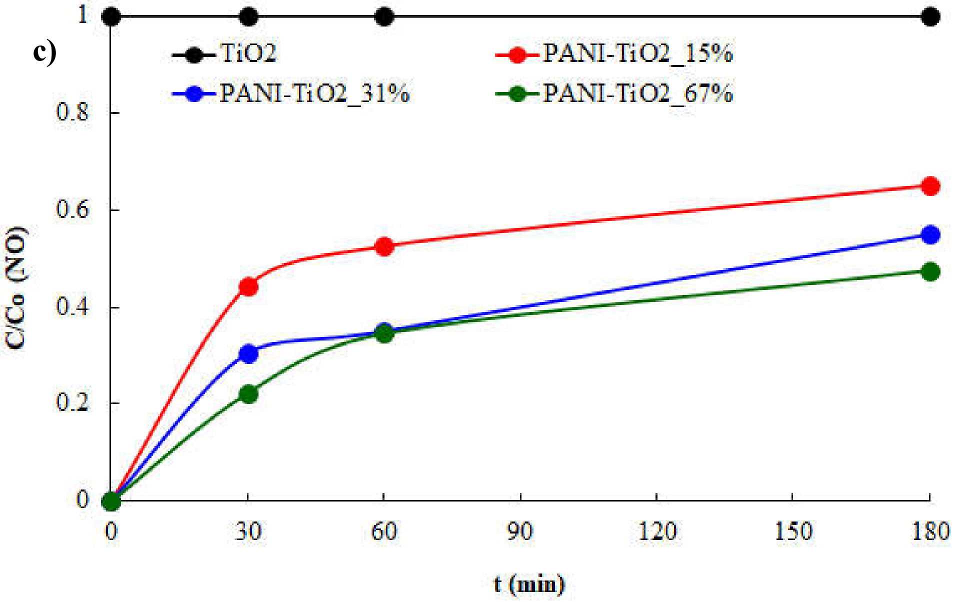

Figure S9: (**a**) NO_2 and (**b**) NOx removal efficiency and (**c**) NO production as a function of time for TiO_2 and PANI/TiO_2 nanocomposites in dark.

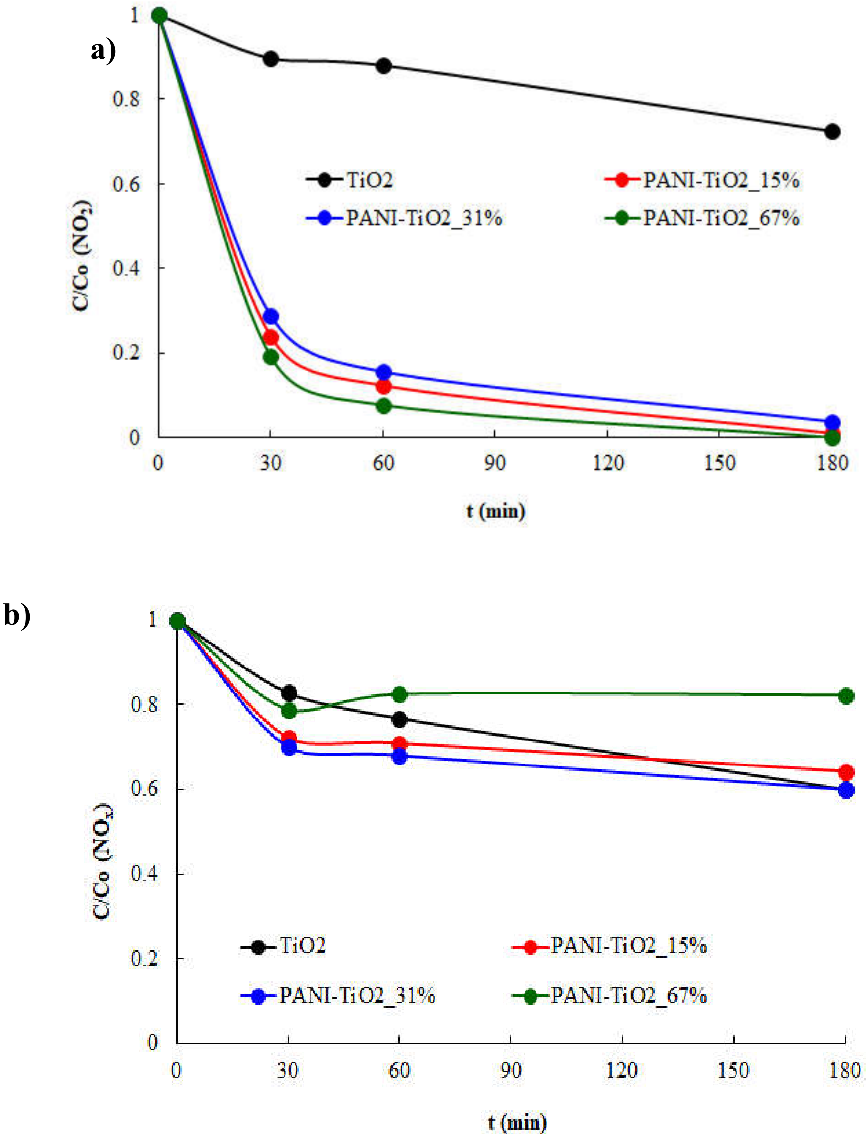

c)

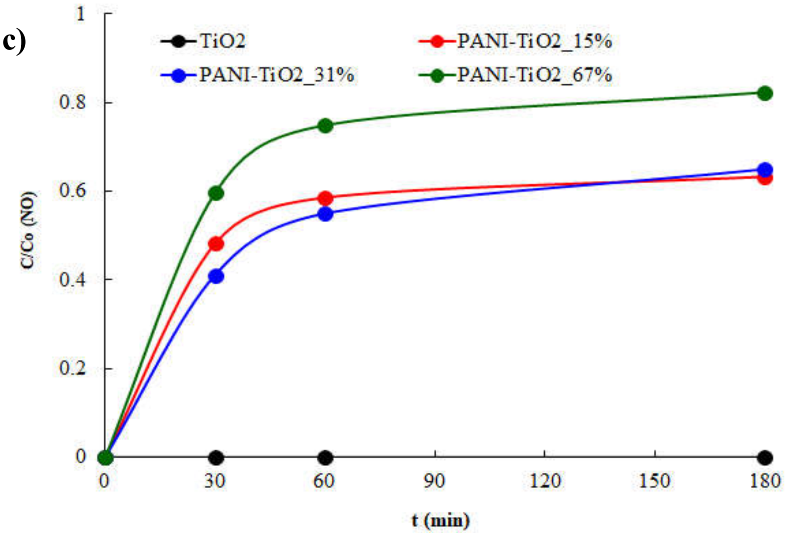

Figure S10: (**a**) NO_2 and (**b**) NOx removal efficiency and (**c**) NO production as a function of time for TiO_2 and PANI/TiO_2 nanocomposites under LED light.

a)

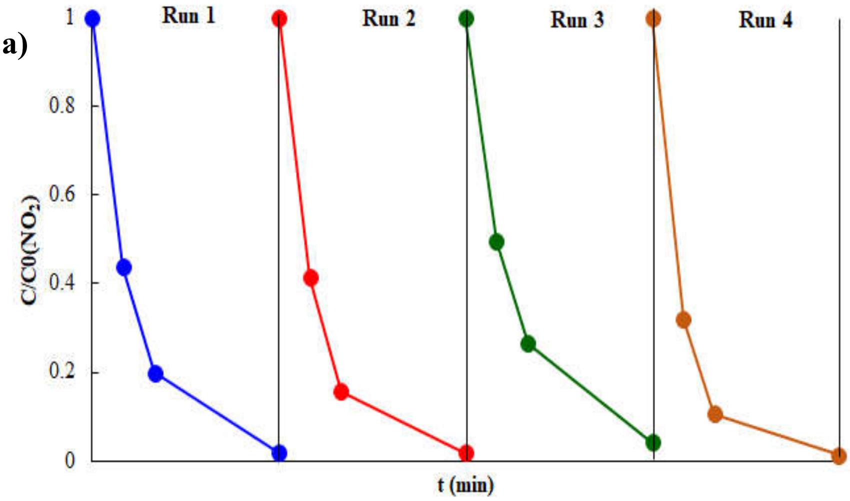

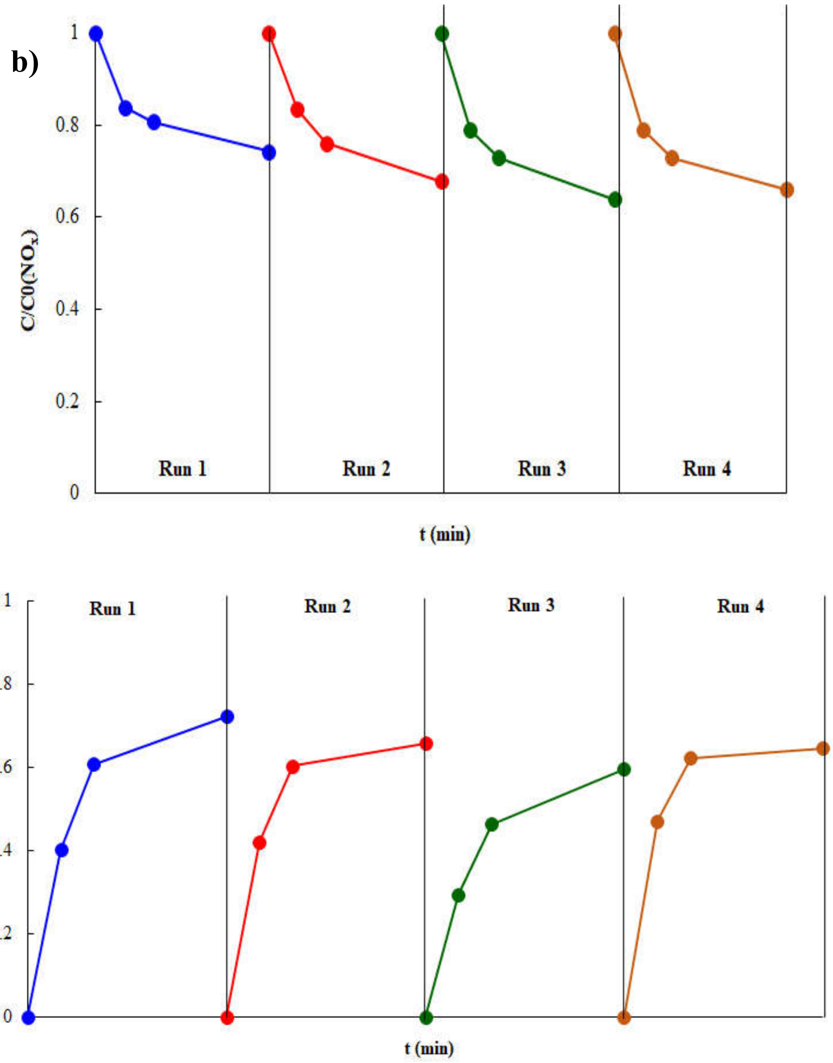

Figure S11: Recycle tests carried out with PANI_ref1 under dark: (**a**) NO_2 and (**b**) NOx removal efficiency and (**c**) NO production.

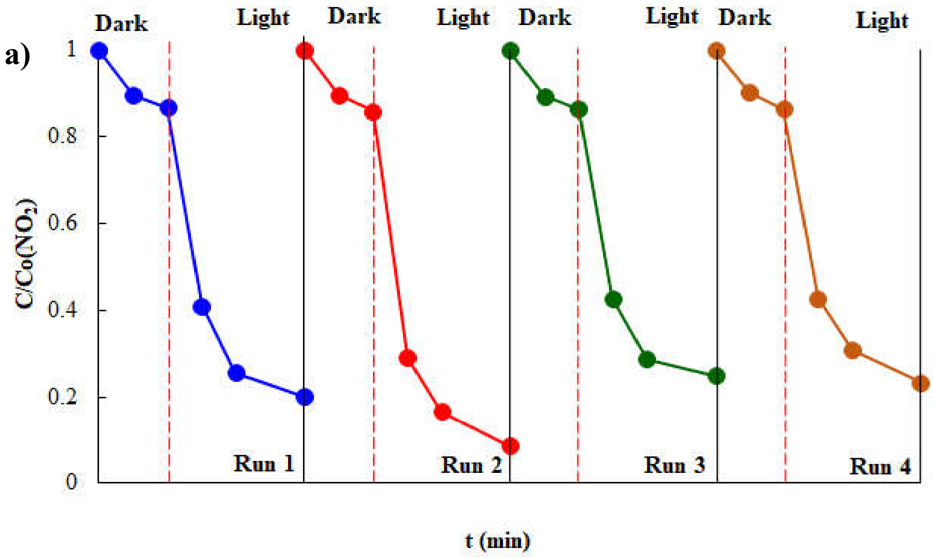

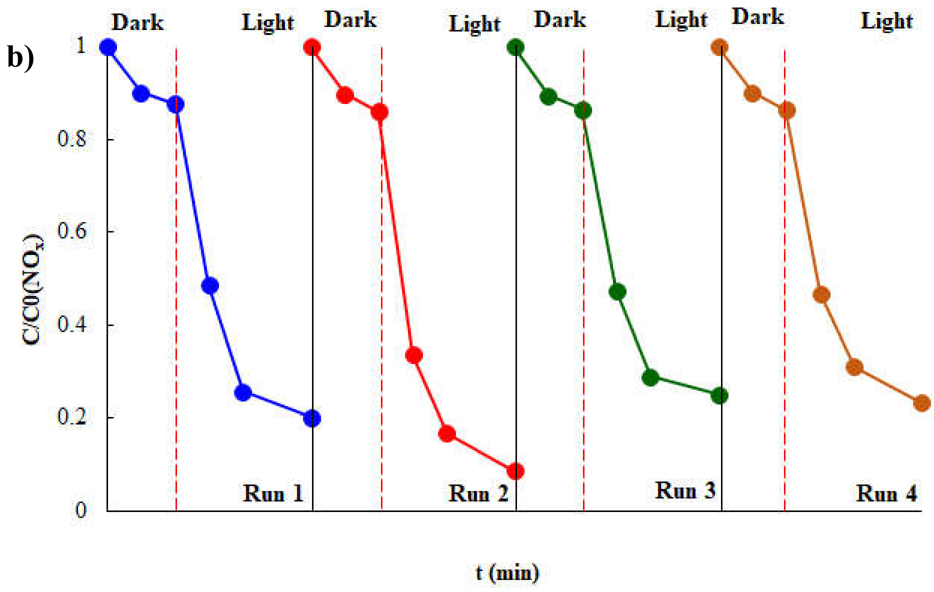

Figure S12: Recycle tests carried out with TiO_2 under UVA irradiation: (**a**) NO_2 and (**b**) NOx removal efficiency.
